# Identification of gene expression profiles associated with prognostic groups of patients with Merkel cell carcinoma

**DOI:** 10.1186/1471-2105-15-S10-P20

**Published:** 2014-09-29

**Authors:** Vinay Raj, Susan Kadlubar

**Affiliations:** 1 Department of Genetics, UAMS, Little Rock, AR 72205, USA

## Background

Merkel cell carcinoma (MCC) is an aggressive form of skin cancer mostly caused by the Merkel cell polyomavirus [1]. MCC although rare in incidence is associated with poor prognosis. Gene expression studies (GES) have identified a number of genes that are associated with risk of developing MCC. However, their clinical utility to predict risk, response to treatment, or treatment toxicity, remains undefined. There is a need to better understand the biology of MCC and to develop potential new targets with regard to cancer risk and prognostic value [2]. Gene expression profiling and pathway association analysis on GES data can elucidate relevant biological processes, and identify new candidate target genes.

## Materials and methods

We sought to identify differential gene expression and functional associations in MCC. Differentially expressed genes with a P value ≤ 0.05 and a fold change ≥ 2.5 between prognostic groups were identified and pathway association analysis was done for a total of 38 differentially expressed genes from a gene expression profiling study [3] with prognostic groups of MCC patients. The over representation of gene-based associations in each pathway was calculated using Fisher's exact test.

## Results

Pathways involved in neuropathic pain signaling, glutamate receptor signaling and synaptic long term potentiation were found to be highly enriched with associations. These results suggest that gene expressions associated with these pathways may contribute to MCC development and prognosis.
